# Supplementary material for: Protease-activated receptor-2 ligands reveal orthosteric and allosteric mechanisms of receptor inhibition
Source: Commun Biol. 2020 Dec 17;3:782. doi: 10.1038/s42003-020-01504-0 (PMC7747594; doi:10.1038/s42003-020-01504-0)
Supplement: Supplementary file 2 — Supplementary Information [file 42003_2020_1504_MOESM2_ESM.pdf]

Protease-activated receptor-2 ligands reveal orthosteric and allosteric mechanisms of receptor inhibition

Kennedy et al.

Supplementary Information

Supplementary Methods.
Tryptase Enzyme Activity Assay
Chemistry Synthetic Routes

Supplementary Figure 1. Inhibition of 2f-LIGRL-NH_2-mediated Ca^{2+} mobilization in CHO-PAR2 cells.

Supplementary Figure 2.
Benzimidazole compounds display biphasic inhibition curves for trypsin activation of PAR2.

Supplementary Figure 3.
AZ3451 displays biphasic inhibition curve for tryptase activation of PAR2 but does not inhibit the tryptase enzyme.

Supplementary Figure 4.
AZ3451 displays biphasic inhibition of trypsin activation of PAR2, independent of interaction with PAR1, cell type or assay format.

Supplementary Figure 5.
Mutagenesis of AZ3451 binding site disrupts biphasic inhibition curve for trypsin activation of PAR2.

Supplementary Figure 6. Negative allosteric modulation of Ca^{2+} mobilization by AZ3451 in CHO-hPAR2 cells.

Supplementary Figure 7.
Administration of PAR2 agonist 2fLIGRLO-NH_2 had no effect on the number of ED1 +ve macrophages in rat paw tissue after 30 min.

Supplementary Figure 8. Alternative delivery modes for AZ8838 and AZ3451.

Supplementary Table S1. β-arrestin recruitment of AZ2429 to PAR1 (F2R), PAR2 (F2RL1) and PAR4 (F2RL3) (DiscoverX profiling service).

Supplementary Table S2. Calculated values of E_{max}, EC_{50} and relative activity for 2f-LIGRL-NH_2 and trypsin in the presence of AZ3451 in CHO-hPAR2 cells.

References

Supplementary Methods

Tryptase Enzyme Activity Assay. The tryptase assay was carried out using beta-tryptase (uniprot P20231) produced in *Pichia pastoris* and using Tos-Gly-Pro-Lys-pNA as substrate (25 mM stock in ethanol) in 100 mM Tris pH 8.0, 100 mM NaCl, 10% glycerol, 0.5 mg mL^{-1} BSA, 0.1% DDM. Substrate (250 µM) was pre-incubated with test compound (10 µM) in assay buffer (200 ul) in clear bottom 96 well Greiner plates at room temperature. The reaction was started by the addition of tryptase to a final concentration of 4.7 nM. Conversion was recorded in a plate reader by monitoring the increase of absorption at λ = 405 nm over time. Activity was determined by linear fit of initial rate.

Chemistry Synthetic Routes.

Preparation of AZ2429: 1-Benzyl-*N*-((S)-3-cyclohexyl-1-(((-4-(methylcarbamoyl)-1,2,3,4-tetrahydronaphthalen-1-yl)amino)-1-oxopropan-2-yl)-1*H*-1,2,3-triazole-4-carboxamide

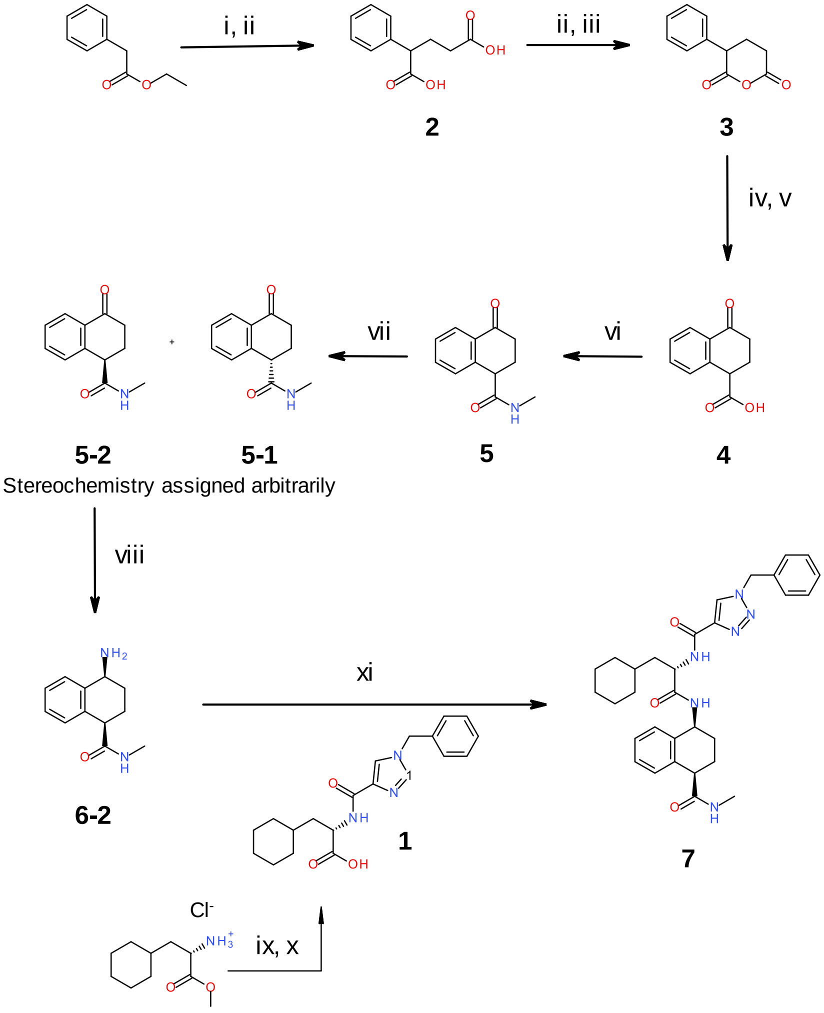

(i) LDA, THF, methyl 3-bromopropanoate, -78°C, 33%; (ii) NaOH, H_2O/MeOH, 25°C, 72%; (iii) Ac_2O, 100°C, 97%; (iv) c. H_2SO_4, 70°C, then MeOH; (v) NaOH, H_2O/MeOH, 50°C, 102%; (vi) $MeNH_2$, HATU, DIEA, THF/DMF, 25°C, 87%; (vii) chiral chromatography; (viii) HCO_2NH_4, $NaCNBH_3$, MeOH, 25°C, 22%; (ix) 1-Benzyl-1H-1,2,3-triazole-4-carboxylic acid, EDC, DIEA, DCM, 44%; (x) LiOH, THF/H_2O, 25°C, 83%; (xi) HATU, DIEA, THF, 26%.

Note: The assignment of the relative and absolute stereochemistry of **6-1** and **6-2** were made by using Vibrational Circular Dichroism (VCD).

Step 1: (±)-1-Ethyl 5-methyl 2-phenylpentanedioate

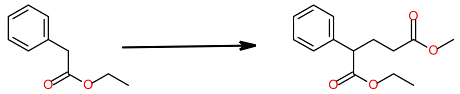

The following reaction was carried out twice on the same scale and then combined for purification:

Lithium diisopropylamide (40.2 mL, 80.4 mmol) was added to ethyl 2-phenylacetate (12 g, 73 mmol) in tetrahydrofuran (200 mL) at -78°C over a period of 10 min under nitrogen. The resulting solution was stirred at -78°C for 1 h. Then methyl 3-bromopropanoate (13.4 g, 80.4 mmol) was added and the resulting solution was stirred at -78°C for 3 h. The reaction mixture was quenched with brine.

The crude reaction mixture from both reactions were combined and then extracted with ethyl acetate (300 mL). The organic layer was dried (Na_2SO_4), filtered and evaporated. The crude product was purified by flash silica chromatography (elution gradient: 20 to 50% ethyl acetate in petroleum ether). Pure fractions were evaporated to dryness to afford (±)-1-ethyl 5-methyl 2-phenylpentanedioate (12 g, 33%) as a pale yellow oil. ^{1}H NMR (400 MHz, DMSO-d_6) δ 7.40 – 7.24 (m, 5H), 4.15 – 4.00 (m, 2H), 3.70 – 3.65 (m, 1H), 3.60 (s, 3H), 2.25 – 2.10 (m, 3H), 2.00 – 1.85 (m, 1H), 1.20 – 1.10 (m, 3H).

Step 2: (±)-2-Phenylpentanedioic acid (2)

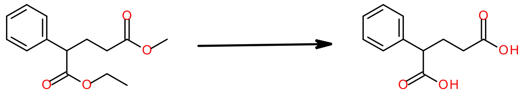

Sodium hydroxide (7.7 g, 0.19 mol) was added in one portion to (±)-1-ethyl 5-methyl 2-phenylpentanedioate (**2**, 12 g, 48 mmol) in methanol (100 mL) and water (50 mL). The resulting mixture was stirred at 25°C for 3 h. The methanol was removed under vacuum, and the reaction mixture was adjusted to pH 5. The aqueous mixture was extracted with ethyl acetate (3×300 mL). The organic layer was dried over anhydrous Na_2SO_4, filtered and evaporated. The crude product was purified by silica chromatography (elution gradient: 0 to 10% methanol in dichloromethane). Pure fractions were evaporated to dryness to afford (±)-2-phenylpentanedioic acid (7.2 g, 72%) as a yellow solid. ^{1}H NMR (400 MHz, DMSO-d_6) δ 12.3 (br s, 2H), 7.40 – 7.20 (m, 5H), 3.60 – 3.50 (m, 1H), 2.20 – 2.00 (m, 3H), 1.90 – 1.75 (m, 1H).

Step 3: (±)-3-Phenyldihydro-2*H*-pyran-2,6(3*H*)-dione (3)

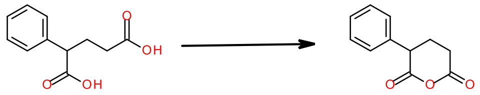

Acetic anhydride (3.53 g, 34.6 mmol) was added to 2-phenylpentanedioic acid (7.2 g, 35 mmol). The resulting mixture was stirred at 100°C for 3 h. The solvent was removed under reduced pressure. The crude product was purified by flash silica chromatography (elution gradient: 0 to 30% ethyl acetate in petroleum ether). Pure fractions were evaporated to dryness to afford (±)-3-phenyldihydro-2*H*-pyran-2,6(3*H*)-dione (6.40 g, 97%) as a pale yellow oil. ^{1}H NMR (400 MHz, DMSO-d_6) δ 7.35 – 7.24 (m, 5H), 4.15 (dd, J =16.0, 8.0 Hz, 1H), 3.03 – 2.80 (m, 2H), 2.45 – 2.31 (m, 1H), 2.06 – 1.97 (m, 1H).

Step 4: (±)-Methyl 4-oxo-1,2,3,4-tetrahydronaphthalene-1-carboxylate

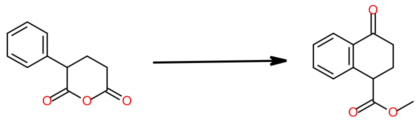

A mixture of 3-phenyldihydro-2*H*-pyran-2,6(3*H*)-dione (6.4 g, 34 mmol) and concentrated sulphuric acid (1.79 mL, 33.7 mmol) was stirred at 70°C for 1.5 h. The resulting mixture was allowed to cool to rt and then added to cooled methanol (100 mL) (ice/water bath) over 15 min. Upon complete addition, the mixture was allowed to warm to rt and stirred for 2 h. The reaction mixture was diluted with ethyl acetate (500 mL) and washed sequentially with saturated brine (2×300 mL). The organic layer was dried over Na_2SO_4, filtered and evaporated to afford crude product. The crude product was purified by flash silica chromatography, (elution gradient: 10 to 30% ethyl acetate in petroleum ether). Pure fractions were evaporated to dryness to afford (±)-methyl 4-oxo-1,2,3,4-tetrahydronaphthalene-1-carboxylate (4.2 g, 61%) as a pale yellow solid. m/z (ES$^+$), $[M+H]^+$ = 205; NH_4HCO_3 pH 10, HPLC tR = 0.77 min.

Step 5: (±)-4-Oxo-1,2,3,4-tetrahydronaphthalene-1-carboxylic acid (4)

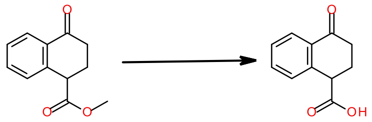

Sodium hydroxide (3.29 g, 82.3 mmol) was added to methyl 4-oxo-1,2,3,4-tetrahydronaphthalene-1-carboxylate (4.2 g, 21 mmol) in methanol (100 mL) and water (50 mL). The resulting mixture was stirred at 50°C for 15 h. After cooling, the reaction mixture was adjusted to pH 5 and then diluted with ethyl acetate (500 mL). The organic extract was washed sequentially with saturated brine (2×300 mL). The organic layer was dried over Na_2SO_4, filtered and evaporated to afford (±)-4-oxo-1,2,3,4-tetrahydronaphthalene-1-carboxylic acid (4.0 g, quantitative yield) as a pale yellow solid. ^{1}H NMR (400 MHz, DMSO-d_6) δ 7.90 – 7.88 (m, 1H), 7.62 – 7.58 (m, 1H), 7.45 – 7.40 (m, 2H), 4.06 – 4.00 (m, 1H), 2.74 – 2.56 (m, 2H), 2.38 – 2.25 (m, 2H).

Step 6: (±)-*N*-Methyl-4-oxo-1,2,3,4-tetrahydronaphthalene-1-carboxamide (5)

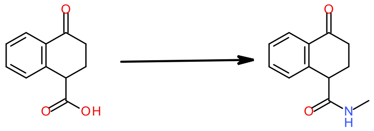

Methylamine (2.0 M in tetrahydrofuran, 11.6 mL, 23.2 mmol) was added to 4-oxo-1,2,3,4-tetrahydronaphthalene-1-carboxylic acid (4.0 g, 21 mmol), HATU (8.8 g, 23 mmol) and di-isopropyl ethyl amine (7.35 mL, 42.1 mmol) in *N*,*N*-dimethyl formamide (100 mL). The resulting solution was stirred at 25°C for 15 h. The reaction mixture was diluted with ethyl acetate (300 mL) and washed sequentially with saturated brine (2×300 mL). The organic layer was dried over Na_2SO_4, filtered and evaporated to afford crude product. The crude product was purified by flash silica chromatography (elution gradient: 20 to 50% ethyl acetate in petroleum ether). Pure fractions were evaporated to dryness to afford (±)-*N*-methyl-4-oxo-1,2,3,4-tetrahydronaphthalene-1-carboxamide (3.7 g, 87%) as a yellow solid. ^{1}H NMR (400 MHz, DMSO-d_6) δ 8.16 (br s, 1H), 7.89 (d, J = 8.0 Hz, 1H), 7.60 – 7.56 (m, 1H), 7.43 – 7.39 (m, 1H), 7.28 (d, J = 8.0 Hz, 1H), 3.86 (d, J = 12.0 Hz, 1H), 2.88 – 2.80 (m, 1H), 2.65 (d, J = 4.0 Hz, 3H), 2.58 – 2.53 (m, 1H), 2.25 – 2.20 (m, 2H).

Step 7: *N*-Methyl-4-oxo-1,2,3,4-tetrahydronaphthalene-1-carboxamide (Enantiomers 5-1 and 5-2)

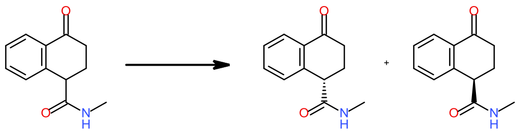

(±)-*N*-methyl-4-oxo-tetralin-1-carboxamide (3.5 g) was resolved by preparative SFC with the following conditions:

Column	ChiralPak-IC-SFC-02, 5 cm×25 cm (5 µm)
Mobile Phase	A: CO_2; B: MeOH
Flow Rate	150 mL/min
Gradient	50% B
Detection	220 nm
Enantiomer 1: Retention Time	3.67
Enantiomer 2: Retention Time	4.65

The fractions containing the desired compound were evaporated to dryness to afford Enantiomer 1 (**5-1**,1.4 g, 40%) as a pale yellow solid. m/z (ES+), $[M+H]^+$ = 204; TFA, HPLC tR = 1.124 min

and Enantiomer 2 (**5-2**, 1.4 g, 40%) as a pale yellow solid. m/z (ES+), $[M+H]^+$ = 204; TFA, HPLC tR = 1.121 min

Chiral Purity was assed using the following conditions:

Column	ChiralPak IC, 3.0×100 mm (3 µM);
Flow Rate	2 mL/min
Mobile Phase	A: CO_2; B: MeOH (0.1 DEA)
Gradient	10% to 50% B over 4 min then hold for 2 min
Detection	220 nm
**5-1** (Enantiomer 1; tR=1.77 min)	100% e.e.
**5-2** (Enantiomer 2; tR=2.30 min)	99.6% e.e.

Step 8: 4-Amino-*N*-methyl-1,2,3,4-tetrahydronaphthalene-1-carboxamide (Enantiomer 6-2)

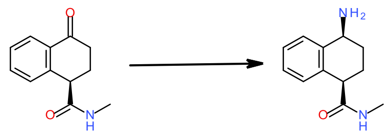

Sodium cyanoborohydride (495 mg, 7.9 mmol) was added in one portion to *N*-Methyl-4-oxo-1,2,3,4-tetrahydronaphthalene-1-carboxamide (**5-2,** 800 mg, 3.9 mmol) and ammonium formate (2.48 g, 39.4 mmol) in methanol (20 mL) at rt. The resulting mixture was stirred at 45°C for 15 h. The crude product was purified by preparative HPLC (Column: XBridge Prep OBD C18 Column 30×150 mm 5um; Mobile Phase A: Water (10 mmol/L NH_4HCO_3 + 0.1% $NH_3.H_2O$), Mobile Phase B: acetonitrile; Flow rate: 60 mL/min; Gradient: 7% 10% B in 8 min; 254/220 nm; Rt: 7.50 min). Fractions containing the desired compound were evaporated to dryness to afford 4-amino-*N*-methyl-1,2,3,4-tetrahydronaphthalene-1-carboxamide (Enantiomer 2, 0.18 g, 22%) as an off-white solid. ^{1}H NMR (400 MHz, Methanol-d_4) δ 7.43 (d, *J* = 7.2 Hz, 1H), 7.30 – 7.16 (m, 2H), 7.09 (d, *J* = 7.6 Hz, 1H) 3.97 (t, *J* = 5.0, 1H), 3.69 (dd, *J* = 8.2, 6.2 Hz 1H), 2.80 (s, 3H), 2.26 – 2.10 (m, 1H), 2.06 – 1.88 (m, 3H). m/z (ES+), 205 = 205; TFA, HPLC tR = 0.84 min. Analysis of 6-2 on chiral HPLC with several methods only revealed a single stereoisomer.

Results of VCD analysis

Calculations

A conformational search was made on the *R*,*S*-configuration of the current analogue (**6**) using MonteCarlo calculations in Maestro (v.10.5.014). The force field OPLS2005 in gas phase with 1000 steps using the PRCG-method was selected. The mixed torsion/low mode method was employed and conformations within 21 kJ/mol from the global minimum were kept. This resulted in four conformations.

A quantum mechanic minimization of the generated structures was performed with Gaussian09 using the level of theory B3LYP/6-31G*. The four low energy conformations were selected as starting point for the analysis. The conformations with <10% distribution was removed and resulted in 2 conformations. In these simulations, an in-house program was used to fit Lorentzian line shapes (12 cm^{-1} line width) to the computed spectra thereby allowing direct comparisons between simulated and experimental spectra. The corresponding simulated spectrum of the *S*,*R* enantiomer was obtained by inversion of the *R*,*S* spectrum.

The same procedure was repeated with the *S,S* configuration which resulted in two conformations after the conformational search and a subsequent Gaussian calculation described above. The corresponding simulated spectrum of the *R,R* enantiomer was obtained by inversion of the *S,S* spectrum.

Experimental

Compound **6-2** (8.2 mg) was dissolved in 90 µL DMSO-d_6. The sample was measured for 6 h as a single block in a 100 µm CaF_2 cell in a Bruker VCD PMA50 spectrometer. The experimental data is shown below (red curve).

Compound **6-1** (8.2 mg) was dissolved in 90 µL DMSO-d_6. The sample (was run for 6 h as a single block in a 100 µm CaF_2 cell in a Bruker VCD PMA50 spectrometer. The experimental data is shown below (blue curve).

Comparison of calculated and experimental data

The experimental VCD data of compound **6-2** (top trace, red) was compared with all possible stereoisomers (not shown). The best alignment was found with the calculated *S,R*-isomer (green) in the range of 1275 – 1100 cm^{-1}. This result suggests that compound **6-2** has a *S,R*-configuration.

Further, the experimental VCD data of compound **6-1** (bottom trace, blue) was compared with all possible stereoisomers (not shown). The best alignment was found with the calculated R,S-isomer (marked in pink) in the range of 1275 – 1100 cm^{-1}. This result suggests that compound **6-1** has *a R,S*-configuration.

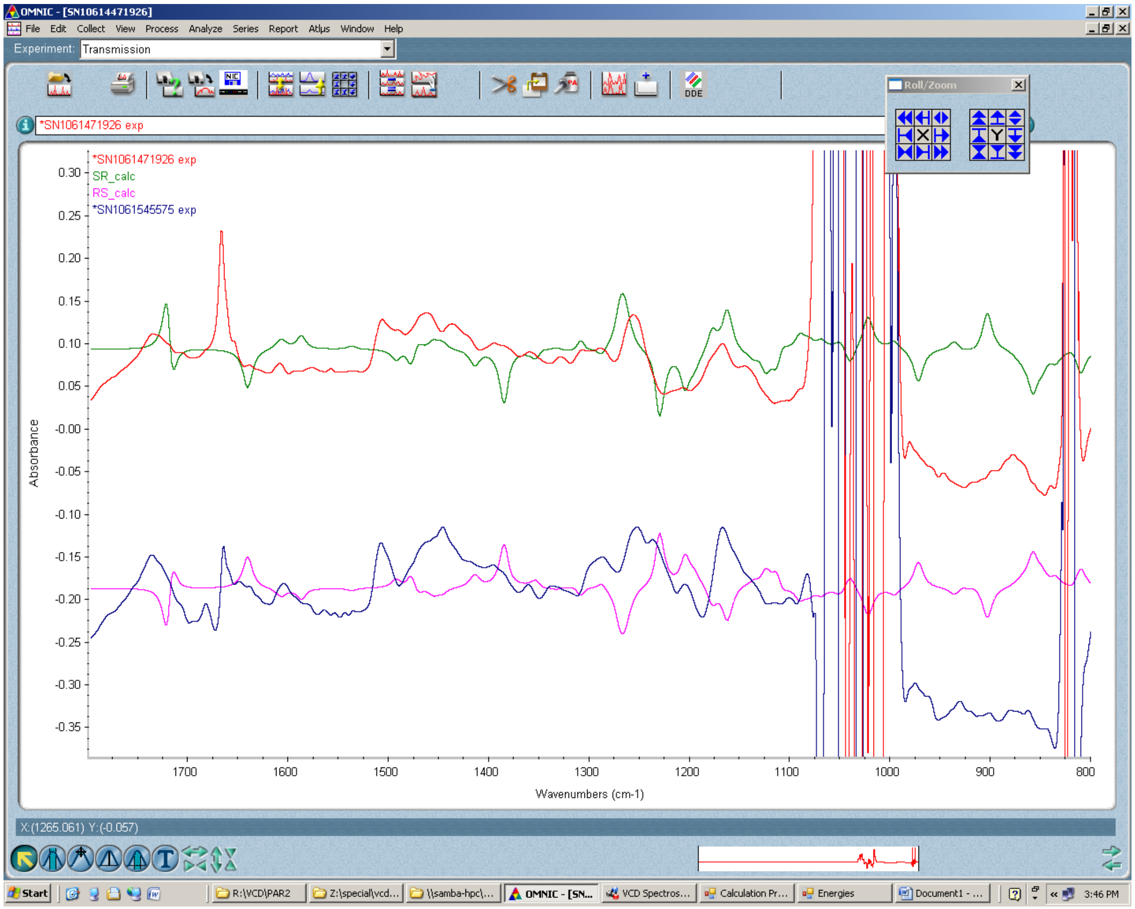

Top: Comparison of experimental VCD data of compound **6-2** (red) with the calculated *S,R*- compound **6-2** (green). Bottom: Comparison of experimental VCD data of compound **6-1** (blue) with calculated *R,S*-compound **6-1** (pink).

Steps 9 and 10: (*S*)-2-(1-Benzyl-1*H*-1,2,3-triazole-4-carboxamido)-3-cyclohexylpropanoic acid (1)

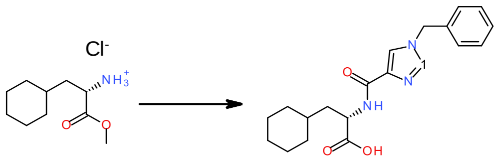

EDC (2.26 g, 11.8 mmol) was added to 1-benzyl-1*H*-1,2,3-triazole-4-carboxylic acid (2.0 g, 9.8 mmol), methyl (*S*)-2-amino-3-cyclohexylpropanoate hydrochloride (2.18 g, 9.8 mmol) and *N*-ethyldiisopropylamine (5.2 mL, 30 mmol) in dichloromethane (30 mL) at 25°C. The resulting mixture was stirred at 25°C for 15 h. The reaction mixture was diluted with dichloromethane (100 mL) and washed with saturated brine. The organic layer was dried over Na_2SO_4, filtered and evaporated to afford crude product. The crude product was purified by flash silica chromatography (elution gradient: 20 to 100% ethyl acetate in petroleum ether). Pure fractions were evaporated to dryness to afford methyl (*S*)-2-(1-benzyl-1*H*-1,2,3-triazole-4-carboxamido)-3-cyclohexylpropanoate (1.6 g, 44%) as a white solid. m/z (ES+), $[M+H]^+$ = 371; TFA, HPLC tR = 1.023 min

Lithium hydroxide (0.39 g, 16 mmol) was added in one portion to methyl (*S*)-2-(1-benzyl-1*H*-1,2,3-triazole-4-carboxamido)-3-cyclohexylpropanoate (1.5 g, 4.1 mmol) in tetrahydrofuran (30 mL) and water (30 mL). The resulting mixture was stirred at 25°C for 15 h. The tetrahydrofuran was removed under reduced pressure. The reaction mixture was adjusted to pH 3. The solid was collected via filtration, washed with water and then dried under vacuum to give (*S*)-2-(1-benzyl-1*H*-1,2,3-triazole-4-carboxamido)-3-cyclohexylpropanoic acid (1.2 g, 83%). ^{1}H NMR (400 MHz, DMSO-d_6): δ 12.6 (s, 1H), 8.67 (s, 1H), 8.50 (d, *J* = 8.0 Hz, 1H), 7.42 – 7.33 (m, 5H), 5.67 (s, 2H), 4.49 – 4.44 (m, 1H), 1.82 – 1.73 (m, 2H), 1.66 – 1.57 (m, 5H),1.38 – 1.30 (m, 1H), 1.22 – 1.05 (m, 3H), 0.98 – 0.79 (m, 2H). m/z (ES^+), $[M+H]^+$ = 357; TFA, HPLC tR = 2.39 min

Step 11: 1-Benzyl-*N*-((*S*)-3-cyclohexyl-1-(((-4-(methylcarbamoyl)-1,2,3,4-tetrahydronaphthalen-1-yl)amino)-1-oxopropan-2-yl)-1*H*-1,2,3-triazole-4-carboxamide (AZ2429)

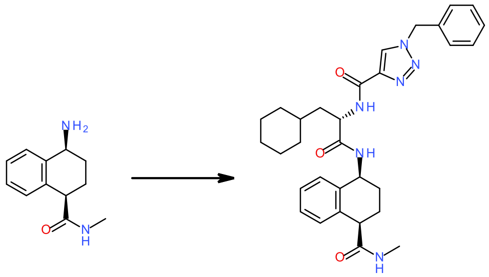

1-[Bis(dimethylamino)methylene]-1*H*-1,2,3-triazolo[4,5-*b*]pyridinium 3-oxid hexafluorophosphate (181 mg, 0.48 mmol) was added to a solution of (*S*)-2-(1-benzyl-1*H*-1,2,3-triazole-4-carboxamido)-3-cyclohexylpropanoic acid (141 mg, 0.40 mmol), and *N*-ethyldiisopropylamine (0.21 mL, 1.2 mmol) in tetrahydrofuran (10 mL) at 25°C. The resulting mixture was stirred at 25°C for 10 min. Then *cis*-4-amino-*N*-methyl-1,2,3,4-tetrahydronaphthalene-1-carboxamide (derived from **5-2**, 81 mg, 0.40 mmol) was added. The resulting mixture was stirred at 25°C for 3 h. The reaction mixture was diluted with water (50 mL) and extracted with ethyl acetate (100 mL). The organic layer was dried over Na_2SO_4, filtered and evaporated to afford crude product. The crude product was purified by preparative HPLC (Column: XBridge Prep OBD C18 Column 30×150mm 5um; Mobile Phase A: Water (10 mmol/L NH_4HCO_3), Mobile Phase B: acetonitrile; Flow rate: 60 mL/min; Gradient: 38% to 65% B in 7 min; 254/220 nm; Rt: 6.30 min). Fractions containing the desired compound were evaporated to dryness to afford 1-benzyl-*N*-((*S*)-3-cyclohexyl-1-(((*1S,4R*)-4-(methylcarbamoyl)-1,2,3,4-tetrahydronaphthalen-1-yl)amino)-1-oxopropan-2-yl)-1*H*-1,2,3-triazole-4-carboxamide (57 mg, 26%) as a white solid. ^{1}H NMR (400 MHz, DMSO-*d*$_6$): δ 8.71 (s, 1H), 8.57 (d, *J* = 8.4 Hz, 1H), 8.28 (d, *J* = 8.4 Hz, 1H), 7.94 – 7.89 (m, 1H), 7.47 – 7.31 (m, 4H), 7.31 – 7.14 (m, 3H), 7.09 – 7.00 (m, 1H), 5.67 (s, 2H), 4.95 – 4.89 (m, 1H), 4.57 – 4.53 (m, 1H), 3.57 (t, *J* = 6.0 Hz, 1H), 2.64 (d, *J* = 4.4 Hz, 3H), 2.08 – 1.82 (m, 3H), 1.79 – 1.52 (m, 8H), 1.30 – 1.05 (m, 4H), 0.98 – 0.75 (m, 2H). m/z (ES$^+$), [M+H]$^+$ = 543; TFA, HPLC tR = 1.91 min

Chiral chromatography showed presence of 4.6% impurity. This is consistent with a small amount of epimerisation during amide formation.

[α]= 0.22 (c = 0.22 g/100 mL in MeOH, T=16).

NOESY spectrum of AZ2429 (see below) revealed NOEs from H4 to H2 (boxed area), indicative that these protons are both axial. The signal for H1 is a triplet (4 Hz each) indicating an equatorial position, and this together identifies the structure of the ring as cis.

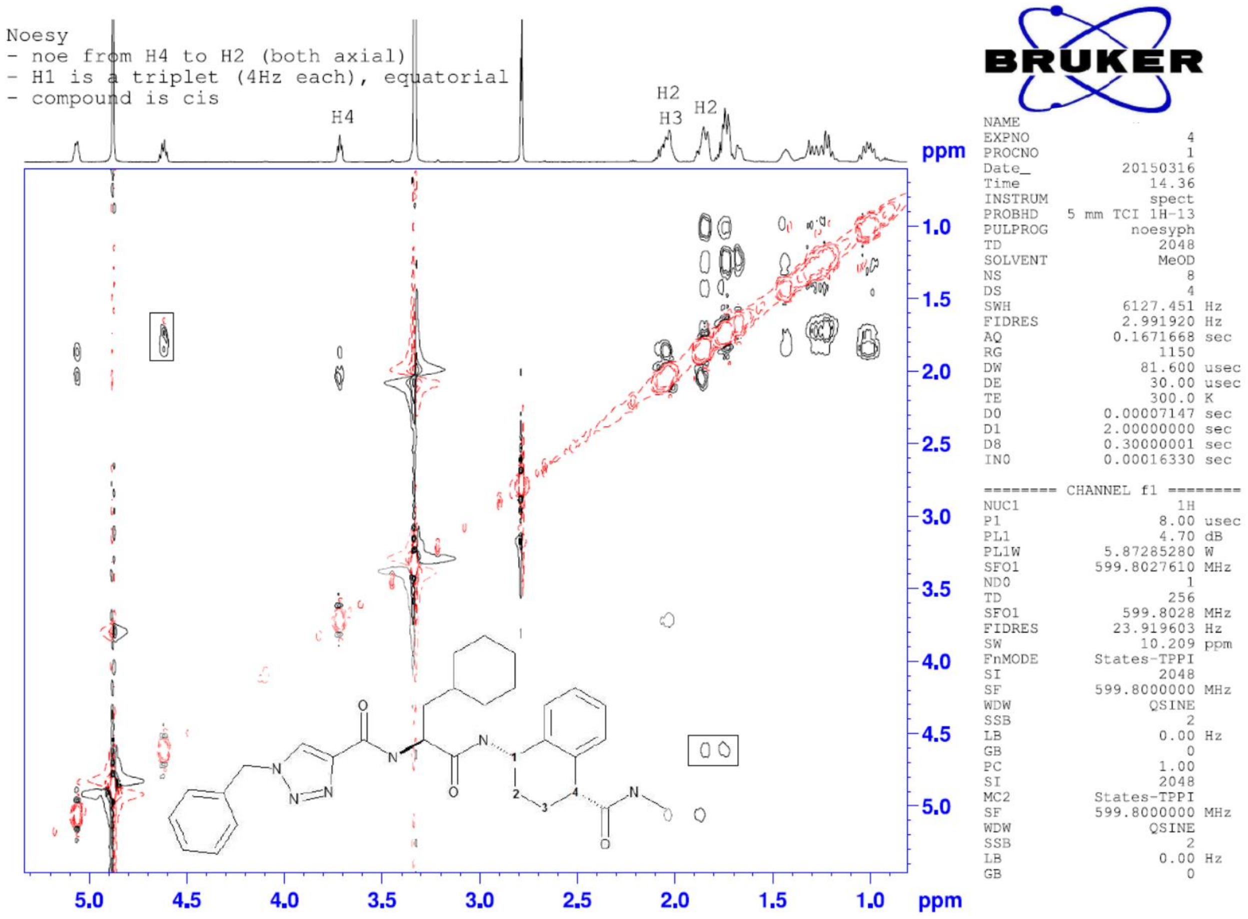

Preparation of AZ2623; 2-(6-Bromo-2*H*-1,3-benzodioxol-5-yl)-1-[(1*S*)-1-cyclohexylethyl]-*N*-(pyridin-4-yl)-1*H*-benzimidazole-5-carboxamide.

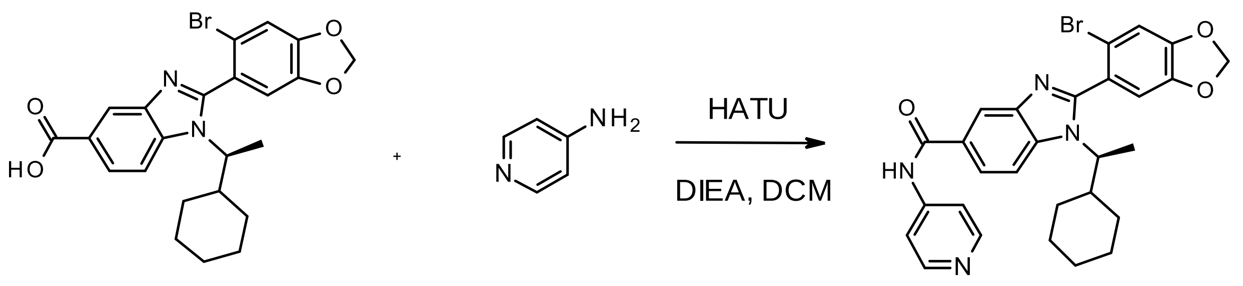

8 **AZ2623**

To a stirred solution of 2-(6-bromo-2*H*-1,3-benzodioxol-5-yl)-1-[(1*S*)-1-cyclohexylethyl]-1*H*-benzimidazole-5-carboxylic acid **8**[2] (300 mg, 0.64 mmol) in dichloromethane (5 mL) was added HATU (484 mg, 1.27 mmol), pyridin-4-amine (90 mg, 0.95 mmol) and *N*-ethyl-*N*-isopropylpropan-2-amine (165 mg, 1.27 mmol). The reaction mixture was stirred at rt for 16 h, dichloromethane was added (30 mL), the mixture washed with water (3 × 30 mL), brine (30 mL), and finally dried over Na_2SO_4. The solution was filtered and concentrated and then purified using Prep-HPLC. The fraction containing the target compound was then concentrated, and dissolved in EtOAc (50 mL). The organic layer was washed with $NaHCO_3$ (sat.) solution, brine, dried over Na_2SO_4, filtered and concentrated to give off-white solid **AZ2623** (191 mg, 55%). ^{1}H NMR (300 MHz, DMSO-*d*$_6$): δ 10.83 – 10.28 (m, 1H), 8.60 – 8.28 (m, 3H), 8.09 – 7.68 (m, 4H), 7.55 – 7.36 (m, 1H), 7.32 – 6.97 (m, 1H), 6.39 – 6.03 (m, 2H), 4.10 – 3.60 (m, 1H), 2.36 – 0.24 (m, 15H). LC-MS $(M+H)^+$ = 547.1, 547.9.

<u>Preparation of AZ8935 : 2-(6-Bromo-2*H*-1,3-benzodioxol-5-yl)-1-[(1*S*)-1-cyclohexylethyl]-*N*-phenyl-1*H*-benzimidazole-5-carboxamide.</u>

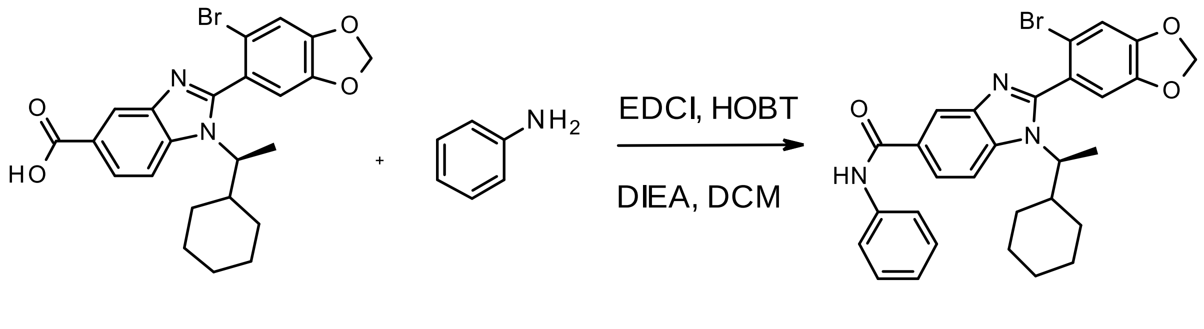

8 **AZ8935**

To a stirred solution of 2-(6-bromo-2*H*-1,3-benzodioxol-5-yl)-1-[(1*S*)-1-cyclohexylethyl]-1*H*-benzimidazole-5-carboxylic acid **8** (80 mg, 0.17 mmol) in dichloromethane (2 mL) was added 1*H*-benzo[*d*][1,2,3]triazol-1-ol hydrate (HOBT) (52 mg, 0.34 mmol) and *N*1-

((ethylimino)methylene)-*N*3,*N*3-dimethylpropane-1,3-diamine hydrochloride (EDCI) (65 mg, 0.34 mmol). To this was added aniline (24 mg, 0.25 mmol) and *N*-ethyl-*N*-isopropylpropan-2-amine (DIEA) (44 mg, 0.34 mmol). The reaction mixture was stirred at room temperature for 16 h, and dichloromethane was added (30 mL). The solution was washed with water (3 × 30 mL), brine (30 mL), dried over Na_2SO_4, filtered and concentrated. The residue was purified on silica gel (Hex/EtOAc; 40+M column) to give a white solid (20 mg, 22%).
^{1}H NMR (300 MHz, DMSO-d_6): δ 10.32 – 9.97 (m, 1H), 8.46 – 8.17 (m, 1H), 7.98 – 7.72 (m, 4H), 7.52 – 7.23 (m, 3H), 7.27 – 6.95 (m, 2H), 6.35 – 6.03 (m, 2H), 3.93 – 3.60 (m, 1H), 2.24 – 0.46 (m, 15H). LC-MS $(M+H)^+$ = 548.1, 549.1.

<u>Preparation of AZ7126: 2-(6-Bromo-2*H*-1,3-benzodioxol-5-yl)-*N*-(4-cyanophenyl)-3-[(1*S*)-1-(oxan-4-yl)ethyl]-3*H*-imidazo[4,5-*b*]pyridine-6-carboxamide.</u>

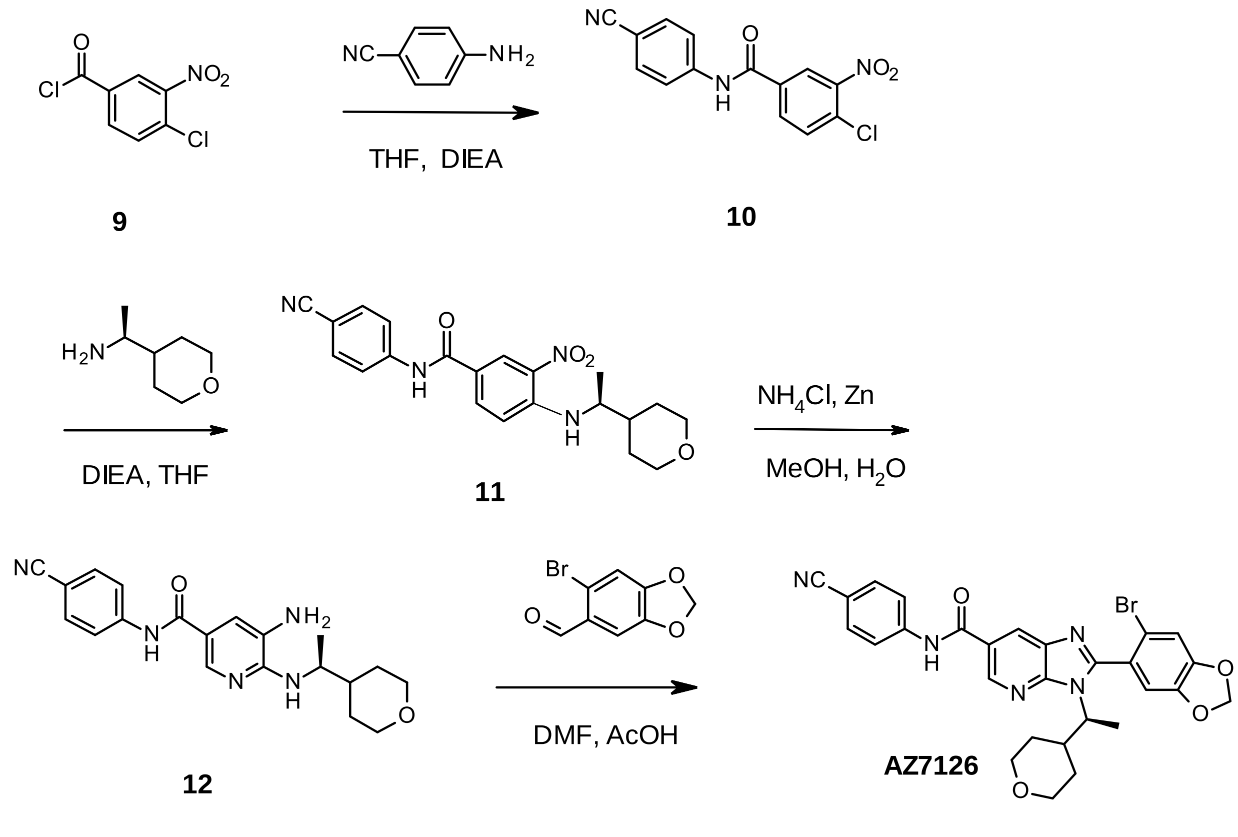

To an ice-cooled solution of 4-chloro-3-nitrobenzoyl chloride **9** (1.0 g, 4.6 mmol) in THF (10 mL) was added 4-aminobenzonitrile (0.591 g, 5.00 mmol), followed by dropwise addition of DIEA

(2.38 mL, 13.6 mmol). The mixture was stirred at room temperature for 1 h and the mixture was carried over to the next step.

Into a 20-mL vial, was placed 4-chloro-*N*-(4-cyanophenyl)-3-nitrobenzamide **10** (1.50 g, 4.97 mmol, 1.00 equiv), (*1S*)-1-(oxan-4-yl)ethan-1-amine hydrochloride (700 mg, 4.23 mmol, 0.80 equiv), tetrahydrofuran (12.5 mL), DIEA (1.62 g, 12.5 mmol, 2.50 equiv). The final reaction mixture was irradiated with microwave radiation for 10 h at 150°C. The reaction was then quenched by the addition of 12.5 mL of water/ice. The resulting solution was extracted with 2 × 15 mL of ethyl acetate and the organic layers combined and dried over anhydrous Na_2SO_4. The resulting solution was diluted with 10 mL of diethyl ether. The solids were collected by filtration. This resulted in *N*-(4-cyanophenyl)-3-nitro-4-[[(1*S*)-1-(oxan-4-yl)ethyl]amino]benzamide (**11**, 1.37 g, 70%) as a yellow solid. LC-MS $(M+H)^+$ = 395.2.

Into a 500-mL 3-necked round-bottom flask, was placed *N*-(4-cyanophenyl)-3-nitro-4-[[(1*S*)-1-(oxan-4-yl)ethyl]amino]benzamide **11** (11.0 g, 27.9 mmol, 1.00 equiv), NH_4Cl, (15.0 g, 280 mmol, 10.1 equiv), methanol (210 mL) and water (40 mL). This was followed by the addition of zinc (14.4 g, 220 mmol, 7.90 equiv) in small batches at 10°C. The resulting solution was stirred for 30 min at 25°C, and the solids were filtered away. The resulting mixture was concentrated in vacuo. The residue was applied to a silica gel column and eluted with dichloromethane/methanol (1:30). This resulted 3-amino-*N*-(4-cyanophenyl)-4-[[(1*S*)-1-(oxan-4-yl)ethyl]amino]benzamide (**12**, 8.7 g, 86%) as a gray foam. LC-MS $(M+H)^+$ = 365.0

To a solution of (*S*)-5-amino-*N*-(4-cyanophenyl)-6-((1-(tetrahydro-2*H*-pyran-4-yl)ethyl)amino)nicotinamide **12** (0.07 g, 0.2 mmol) and 6-bromobenzo[*d*][1,3]dioxole-5-carbaldehyde (0.037 g, 0.16 mmol) in DMF (1 mL) was added 2 drops of AcOH. The resulting solution was stirred at 90°C for 20 h and evaporated. The residue was purified on 12 g silica gel eluted with EtOAc/hexanes (0 – 100%) to give (*S*)-2-(6-bromobenzo[*d*][1,3]dioxol-5-yl)-*N*-(4-cyanophenyl)-3-(1-(tetrahydro-2*H*-pyran-4-yl)ethyl)-3*H*-imidazo[4,5-*b*]pyridine-6-carboxamide **AZ7126** (0.048 g, 49%) as a light yellow solid.

^{1}H NMR (300 MHz, DMSO-d_6) δ 9.40 (br s,1H), 9.04 (s, 1H), 8.91 – 8.70 (m, 1H), 7.91 (d, *J* = 8.5 Hz, 2H), 7.65 (d, *J* = 8.9 Hz, 2H), 7.16 – 6.99 (m, 1H), 6.84 – 6.58 (m, 1H), 6.12 (s, 2H), 4.01 (dd, *J* = 11.1, 3.7 Hz, 1H), 3.81 (d, *J* = 10.4 Hz, 2H), 3.41 (t, *J* = 120 Hz, 1H), 3.28 – 3.09 (m, 1H), 2.87 – 2.62 (m, 1H), 1.91 – 1.63 (m, 4H), 1.15 – 0.72 (m, 2H). LC-MS $(M+H)^+$: = 576.0, 577.1.

AZ3451 was prepared according to the procedure in Cheng et al.[2]

AZ8838 and **AZ0107** were prepared according to the procedure in Cumming et al.[3]

Supplementary Figures

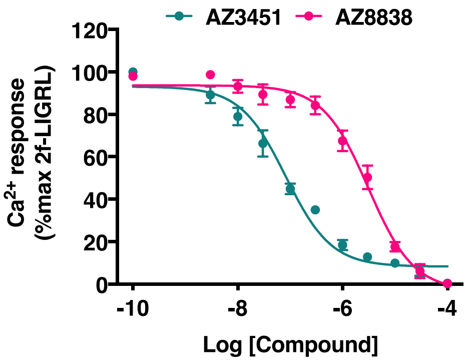

Supplementary Figure 1. Inhibition of 2f-LIGRL-NH_2-mediated Ca^{2+} mobilization in CHO-PAR2 cells. AZ3451 (cyan) and AZ8838 (magenta) were pre-incubated for 60 min 37 °C followed by the addition of 2f-LIGRL-NH_2 (1µM). Data presented as mean ± s.e.m of n ≥ 3 independent experiments.

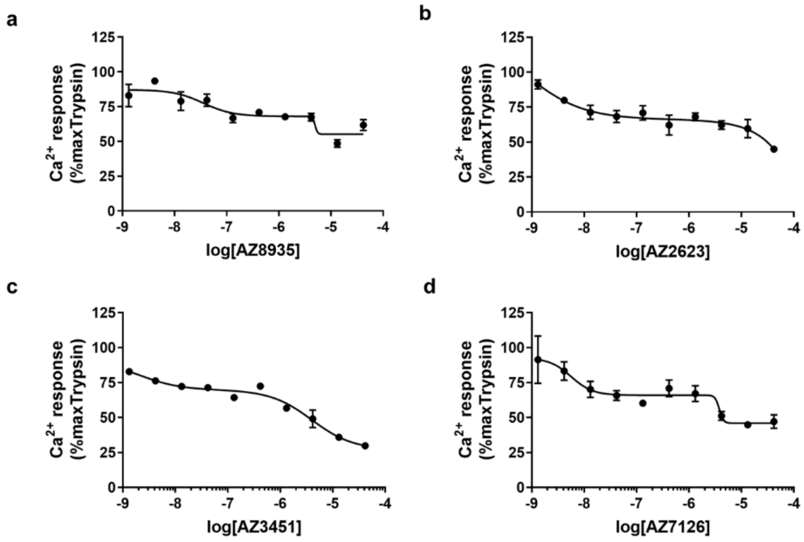

Supplementary Figure 2. Benzimidazole compounds display biphasic inhibition curves for trypsin activation of PAR2. The trypsin-induced Ca^{2+} response was measured in 1321N1-hPAR2 cells in the presence of 1 µM Vorapaxar to silence PAR1 activation. All benzimiazole compounds **a** AZ8935, **b** AZ2623, **c** AZ3451 or **d** AZ7126 displayed only partial inhibition at low

nanomolar concentrations, with further inhibition in the low micromolar range. Data are presented as mean ± s.e.m. based n=3 independent experiments.

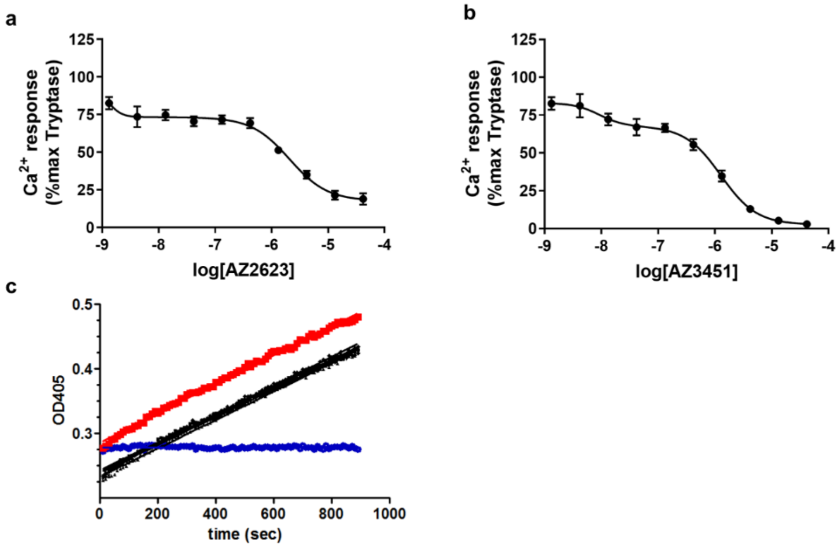

Supplementary Figure 3. AZ3451 displays biphasic inhibition for tryptase activation of PAR2 but does not inhibit the tryptase enzyme. The tryptase-induced Ca^{2+} response was measured in 1321N1-hPAR2 cells in the presence of 1 µM Vorapaxar to silence PAR1 activation. The benzimidazole compounds **a** AZ2623 and **b** AZ3451 displayed only partial inhibition at low nanomolar concentrations, with further inhibition at low micromolar concentrations. Data are presented as mean ± s.e.m., n=9 independent experiments. **c** Tryptase activity was completely inhibited by 10 µM of tryptase inhibitor, 4-nitrophenyl-4-guanidinobenzoate (blue), whereas no inhibition was detected for 10 µM AZ8838 or AZ3451 (black), which had a similar slope to the DMSO control (red) (n=1). Method can be found in the Supplementary materials.

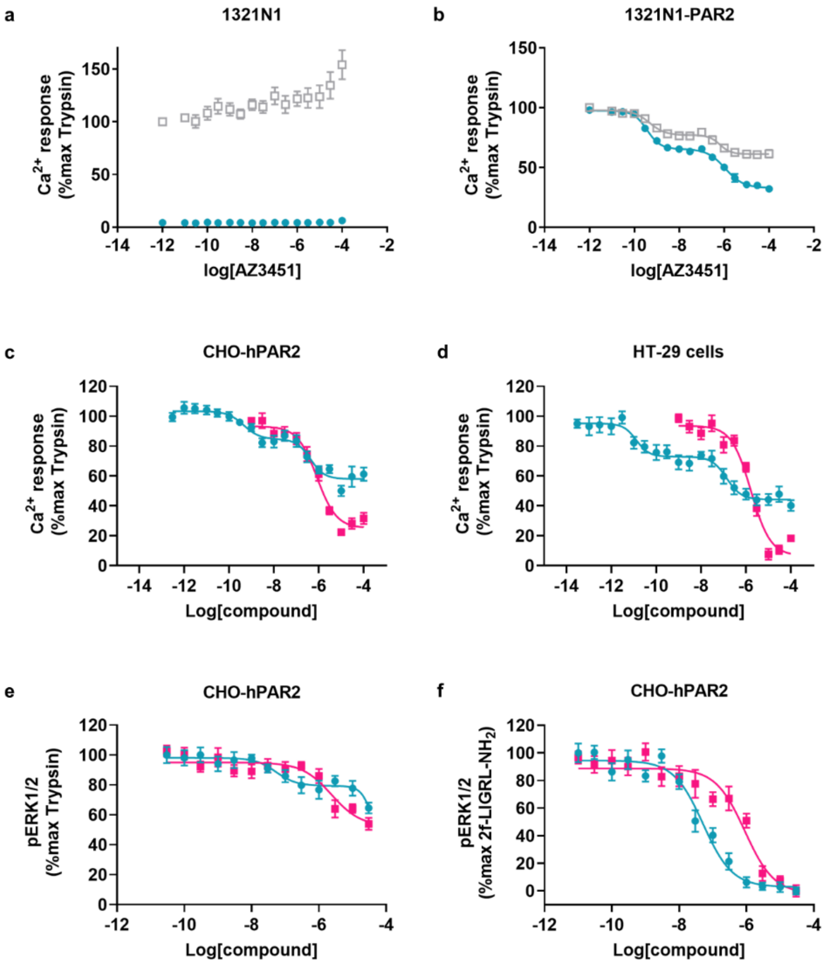

Supplementary Figure 4. AZ3451 displays biphasic inhibition of trypsin-mediated PAR2 activation, independent of interaction with PAR1, cell type or assay format. **a** AZ3451 appears to potentiate trypsin-induced activation of PAR1 (grey), which is endogenously expressed in 1321N1 cells. This effect was completely blocked when trypsin activation was measured in the presence of PAR1 antagonist vorapaxar (cyan) which was included in subsequent experiments on this cell line (n=3). **b** the biphasic nature of AZ3451 inhibition of trypsin-mediated PAR2 activation is independent of interaction with PAR1 as seen by the similar response detected with (cyan) and without (grey) addition of vorapaxar (n=4). A similar biphasic inhibitory response of AZ3451 (cyan) was seen for trypsin-induced activation of Ca^{2+} signalling via PAR2, in **c** CHO-hPAR2 (n=4) and **d** HT-29 cells (n=3), as well as when monitoring **e** trypsin-mediated phosphorylation of ERK1/2 via PAR2 (n=3). For comparison the monophasic inhibitory curves of trypsin-activation by AZ8838 (magenta) are included. **f** Monophasic inhibition by AZ3451 (cyan) and AZ8838 (magenta) of peptide-mediated phosphorylation of ERK1/2 via PAR2 was seen in CHO-hPAR2 cells (n=3). Antagonist responses were calculated as % inhibition of agonist response in the absence of antagonist. Data are presented as mean ± s.e.m. of n independent experiments.

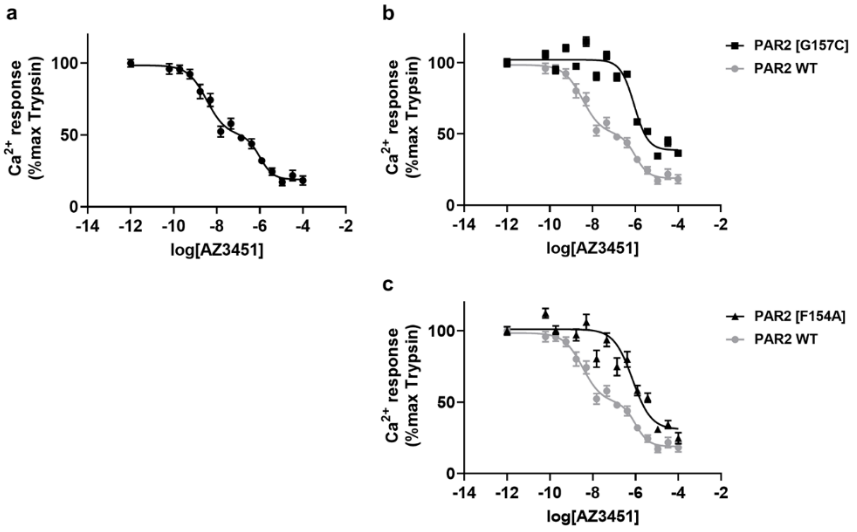

Supplementary Figure 5. Mutagenesis of the AZ3451 binding site disrupts biphasic inhibition of trypsin-mediated PAR2 activation. The trypsin-induced Ca^{2+} response was measured in 1321N1 cells transiently transfected with human PAR2 either **a** wild type or with one single point mutation **b** [G157C] or **c** [F154A], in the presence of 1 µM Vorapaxar to silence PAR1 activation. Antagonist responses were calculated as % inhibition of trypsin response in the absence of antagonist. Data are presented as mean ± s.e.m. of n=4 independent experiments.

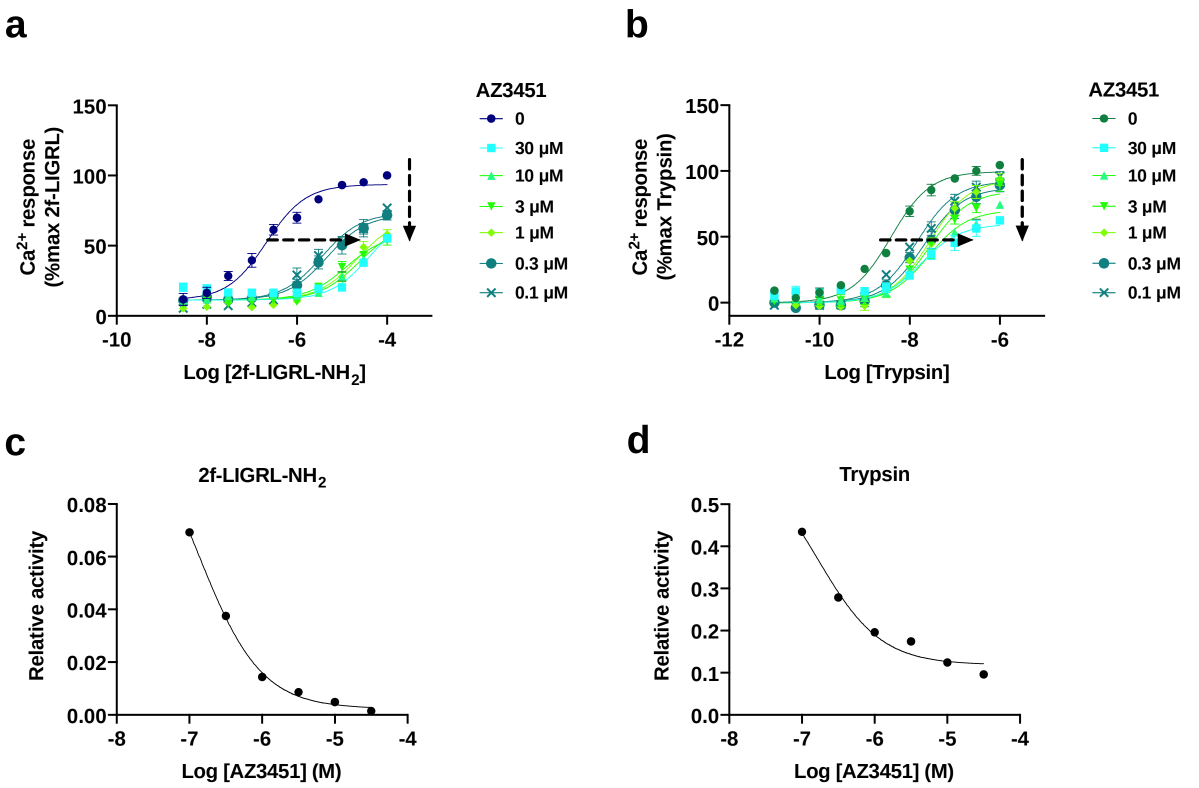

Supplementary Figure 6. Negative allosteric modulation of Ca^{2+} mobilization by AZ3451 in CHO-hPAR2 cells. Concentration response curves for (a) 2f-LIGRL-NH_2 or (b) trypsin were measured in the presence of increasing concentrations of AZ2341. Curves were fitted according to the operational model of allosterism and AZ4351 exhibits a classical rightward and downward curve shifts of a negative allosteric modulator ($\alpha < 1$) against both 2f-LIGRL-NH_2 and trypsin. Data presented as mean ± s.e.m of n ≥ 3 independent experiments. Relative activity of (c) 2f-LIGRL-NH_2 or (d) trypsin, calculated from Supplementary Table 1 using Ca^{2+} mobilisation in the presence of increasing concentrations of AZ2341. Curves were fitted using a 4-parameter fit and data presented as mean of n ≥ 3 independent experiments.

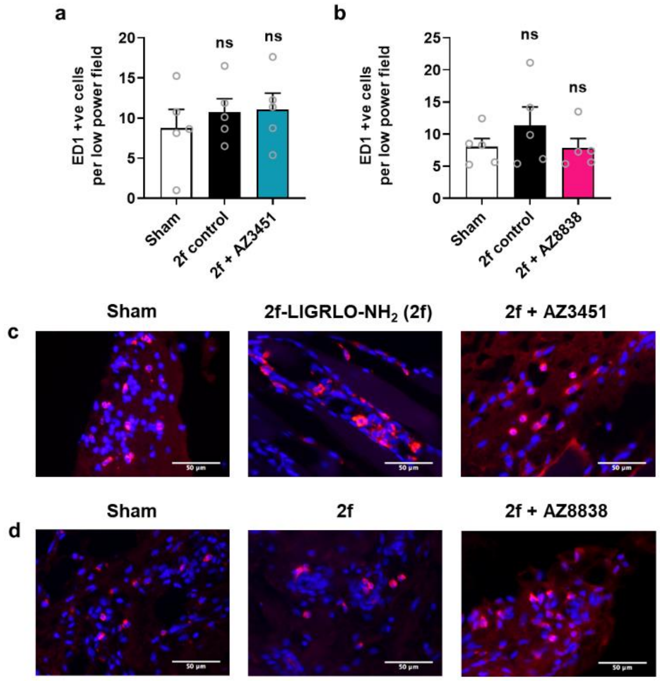

Supplementary Figure 7. PAR2-induced inflammation did not alter ED1-positive macrophage levels. Administration of PAR2 agonist 2fLIGRLO-NH_2 had no effect on the number of ED1-positive (ED1 +ve) macrophages in rat paw tissue after 30 min. Administration with **a** AZ3451 or **b** AZ8838 also gave no significant difference in number of ED1 +ve cells in rat paw tissue at 30 min after agonist administration compared to sham. Immunohistochemistry images for **c** AZ3451 and **d** AZ8838, ED1 +ve macrophages are stained red, nuclei are stained blue by DAPI. Data are presented as mean ± s.e.m., n=5 male Wistar rats (7–8 weeks old). Statistical analysis by one-way ANOVA.

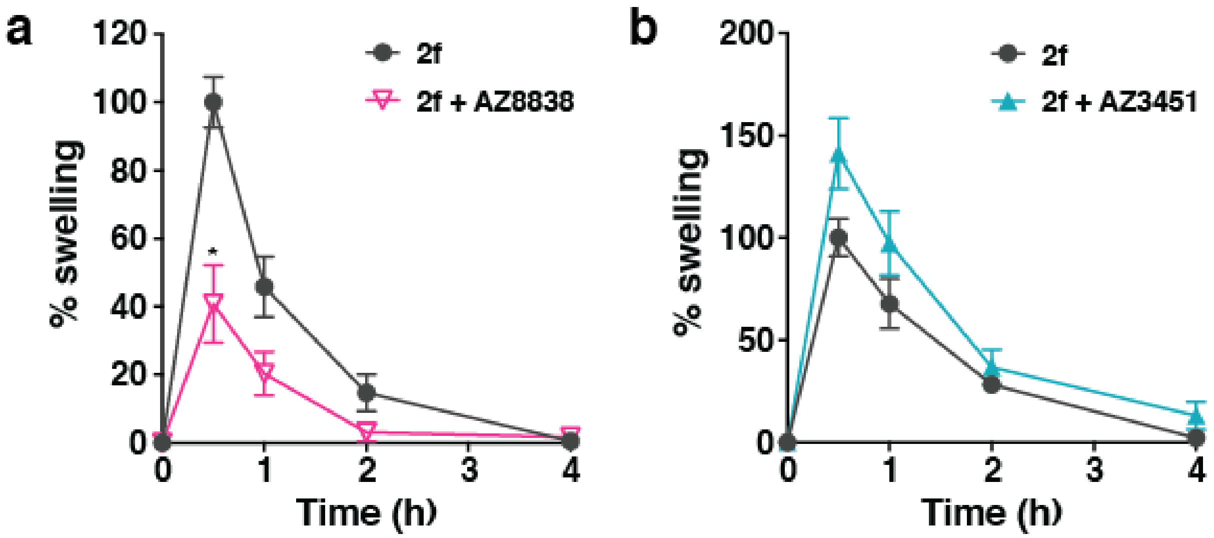

Supplementary Figure 8. Alternative delivery modes for AZ8838 and AZ3451. a PAR2 agonist 2f-LIGRLO-NH_2 (2f, 350 µg/paw, i.pl.) caused peak paw swelling at 30 min. AZ8838 (10 mg kg^{-1}) administered subcutaneously 30 min before 2f-LIGRLO-NH_2 significantly reduced paw swelling. **b** This was not the case for AZ3451 (10 mg kg^{-1}) when administered orally 2 h before 2f-LIGRLO-NH_2.

Supplementary Table S1. β-arrestin recruitment of AZ2429 to PAR1 (F2R), PAR2 (F2RL1) and PAR4 (F2RL3) (DiscoverX profiling service).

Compound Name	Assay Name	Assay Format	Assay Target	Result Type	RC50 (μM)	Hill	Curve Bottom	Curve Top	Max Response
**AZ2429**	Arrestin	Agonist	F2R	EC50	>50				0
**AZ2429**	Arrestin	Antagonist	F2R	IC50	>50				0
**AZ2429**	Arrestin	Agonist	F2RL1	EC50	0.025	1.29	-2	103.3	104.3
**AZ2429**	Arrestin	Antagonist	F2RL1	IC50	>50				4.5
**AZ2429**	Arrestin	Agonist	F2RL3	EC50	>50				3.8
**AZ2429**	Arrestin	Antagonist	F2RL3	IC50	>50				2.3
**TFLLR-NH2**	Arrestin	Agonist	F2R	EC50	3.19	0.87	0.7	100	103.2
**SLIGRL-NH2**	Arrestin	Agonist	F2RL1	EC50	0.68	1.25	0.6	99.9	104.9
**AYPGKF-NH2**	Arrestin	Agonist	F2RL3	EC50	8.62	1.59	-0.2	100.8	102.2

Supplementary Table S2. Calculated values of E_{max}, EC_{50} and relative activity for 2f-LIGRL-NH_2 and trypsin in the presence of AZ3451 in CHO-hPAR2 cells.

**Agonist**	**AZ3451 (μM)**	**E_{max}**	**EC_{50} (μM)**	**Relative activity**
2f-LIGRL-NH_2	30	55	94.6	0.002
	10	55	28.4	0.005
	3	53	15.4	0.009
	1	58	10.1	0.01
	0.3	72	4.8	0.04
	0.1	72	2.6	0.07
	0	100	0.25	1
**Agonist**	**AZ3451 (μM)**	**E_{max}**	**EC_{50} (μM)**	**Relative activity**
Trypsin	30	61	0.038	0.09
	10	74	0.035	0.12
	3	89	0.030	0.17
	1	90	0.027	0.20
	0.3	90	0.019	0.28
	0.1	96	0.013	0.43
	0	102	0.006	1

References

1 Freedman, T. B., Cao, X., Dukor, R. K. & Nafie, L. A. Absolute configuration determination of chiral molecules in the solution state using vibrational circular dichroism. *Chirality* **15**, 743–758 (2003).

2 Cheng, R. K. Y. *et al.* Structural insight into allosteric modulation of protease-activated receptor 2. *Nature* **545**, 112–115, doi:10.1038/nature22309 (2017).

3 Cumming, J. G. *et al.* Preparation of substituted imidazole and triazole compounds as inhibitors of protease-activated receptor-2. WO/2017/194716 (2017).
