# Supplementary material for: Protease-activated receptor-2 ligands reveal orthosteric and allosteric mechanisms of receptor inhibition
Source: Commun Biol. 2020 Dec 17;3:782. doi: 10.1038/s42003-020-01504-0 (PMC7747594; doi:10.1038/s42003-020-01504-0)
Supplement: Supplementary file 3 — Description of Supplementary Files [file 42003_2020_1504_MOESM3_ESM.docx]

**Description of Additional Supplementary Files**

**File Name:** Supplementary Data 1

**Description:** Source data for figures.
