# Supplementary material for: Protease-activated receptor-2 ligands reveal orthosteric and allosteric mechanisms of receptor inhibition
Source: Commun Biol. 2020 Dec 17;3:782. doi: 10.1038/s42003-020-01504-0 (PMC7747594; doi:10.1038/s42003-020-01504-0)
Supplement: Supplementary file 5 — Reporting Summary [file 42003_2020_1504_MOESM5_ESM.pdf]

nature research

Corresponding author(s): Niek Dekker

Last updated by author(s): Oct 19, 2020

Reporting Summary

Nature Research wishes to improve the reproducibility of the work that we publish. This form provides structure for consistency and transparency in reporting. For further information on Nature Research policies, see Authors & Referees and the Editorial Policy Checklist .

Statistics

For all statistical analyses, confirm that the following items are present in the figure legend, table legend, main text, or Methods section.

n/a	Confirmed	
☐	☒	The exact sample size (*n*) for each experimental group/condition, given as a discrete number and unit of measurement
☐	☒	A statement on whether measurements were taken from distinct samples or whether the same sample was measured repeatedly
☐	☒	The statistical test(s) used AND whether they are one- or two-sided<br>*Only common tests should be described solely by name; describe more complex techniques in the Methods section.*
☒	☐	A description of all covariates tested
☐	☒	A description of any assumptions or corrections, such as tests of normality and adjustment for multiple comparisons
☐	☒	A full description of the statistical parameters including central tendency (e.g. means) or other basic estimates (e.g. regression coefficient) AND variation (e.g. standard deviation) or associated estimates of uncertainty (e.g. confidence intervals)
☐	☒	For null hypothesis testing, the test statistic (e.g. *F*, *t*, *r*) with confidence intervals, effect sizes, degrees of freedom and *P* value noted<br>*Give P values as exact values whenever suitable.*
☒	☐	For Bayesian analysis, information on the choice of priors and Markov chain Monte Carlo settings
☒	☐	For hierarchical and complex designs, identification of the appropriate level for tests and full reporting of outcomes
☒	☐	Estimates of effect sizes (e.g. Cohen's *d*, Pearson's *r*), indicating how they were calculated

Our web collection on statistics for biologists contains articles on many of the points above.

Software and code

Policy information about availability of computer code

Data collection	Data were collected using the corresponding software provided on the commercially available instruments (as stated in the Methods)
Data analysis	Data were analyzed using GraphPad (v7 or v8) or Genedata Screener

For manuscripts utilizing custom algorithms or software that are central to the research but not yet described in published literature, software must be made available to editors/reviewers. We strongly encourage code deposition in a community repository (e.g. GitHub). See the Nature Research guidelines for submitting code & software for further information.

Data

Policy information about availability of data

All manuscripts must include a data availability statement. This statement should provide the following information, where applicable:

- Accession codes, unique identifiers, or web links for publicly available datasets
- A list of figures that have associated raw data
- A description of any restrictions on data availability

The authors declare that the data supporting the findings of this study are available within the article and its Supplementary Information files. All the other data supporting the findings of this study are available from the corresponding author upon reasonable request.

Field-specific reporting

Please select the one below that is the best fit for your research. If you are not sure, read the appropriate sections before making your selection.

☒ Life sciences ☐ Behavioural & social sciences ☐ Ecological, evolutionary & environmental sciences

Life sciences study design

All studies must disclose on these points even when the disclosure is negative.

Sample size	No sample-size calculations were performed. Sample size was determined to be adequate based on the magnitude and consistency of measurable differences between groups.
Data exclusions	No data were excluded
Replication	Replicate experiments were successful. Data included in this manuscript are from two independent laboratories, and were reproducible across both.
Randomization	Rats analyzed were litter mates and sex-matched, and allocated into experimental groups at random.
Blinding	Investigators were not blinded to compounds additions during experiments. Data reported for experiments are not subjective but rather based on quantitative measurements.

Reporting for specific materials, systems and methods

We require information from authors about some types of materials, experimental systems and methods used in many studies. Here, indicate whether each material, system or method listed is relevant to your study. If you are not sure if a list item applies to your research, read the appropriate section before selecting a response.

Materials & experimental systems

n/a	Involved in the study	
☐	☒	Antibodies
☐	☒	Eukaryotic cell lines
☒	☐	Palaeontology
☐	☒	Animals and other organisms
☒	☐	Human research participants
☒	☐	Clinical data

Methods

n/a	Involved in the study	
☒	☐	ChIP-seq
☒	☐	Flow cytometry
☒	☐	MRI-based neuroimaging

Antibodies

Antibodies used	Primary antibodies for tryptase (AA1, ab2378, Abcam, Australia), histamine (H7403, Sigma-Aldrich, USA), neutrophil elastase (ab21595, Abcam, Australia) and ED1 (ab31630, Abcam, Australia) were purchased from commercial sources
Validation	Validation from manufacturer's websites:<br>ab2378 – Validated in WB, ELISA, IHC, ICC, ICC/IF and tested in Mouse, Rat, Human, Cited in 52 publications. H7403 – The product specifically stains histamine-containing cells in paraformaldehyde/carbodiimide-fixed or paraformaldehyde-fixed, paraffin-embedded sections of rat stomach (endocrine cells and mast cells), reacts with a wide range of species, cited in 8 publications. ab21595 – Suitable for: IHC-Fr, ICC/IF, ICC, IHC-P, WB, IP, ELISA, reacts with: Mouse, Rat, Human, cited by 69 publications. ab31630 – Suitable for: Flow Cyt, IHC-Fr, IP, WB, RIA, IHC-FrFl, IHC-P, reacts with: Mouse, Rat, Human, cited by 157 publications. Further information available on manufacturer's websites.

Eukaryotic cell lines

Policy information about cell lines

Cell line source(s)	1321N1 and HT-29 cells were from ECACC; U2OS-hPAR2 were from DiscoverX; rat KNRK cells were from ATCC. More information in Methods section.
Authentication	Short tandem repeat analysis was used to confirm the identity of the cell lines.
Mycoplasma contamination	All cells used tested negative for mycoplasma.
Commonly misidentified lines (See ICLAC register)	No cell lines used are listed in this database.

Animals and other organisms

Policy information about studies involving animals; ARRIVE guidelines recommended for reporting animal research

Laboratory animals	Male Wister rats (7–8 weeks old, 250 ± 30 g)
Wild animals	Study did not involve wild animals
Field-collected samples	Study did not involve samples collected from the field.
Ethics oversight	All in vivo experimental procedures and animal handling were conducted with approval from the Animal Ethics Committee at the University of Queensland which follows NHMRC and ARRIVE guidelines.

Note that full information on the approval of the study protocol must also be provided in the manuscript.
